# Tissue Specific Impacts of a Ketogenic Diet on Mitochondrial Dynamics in the BTBR^T+tf/j^ Mouse

**DOI:** 10.3389/fphys.2016.00654

**Published:** 2016-12-27

**Authors:** Christopher Newell, Timothy E. Shutt, Younghee Ahn, Dustin. S. Hittel, Aneal Khan, Jong M. Rho, Jane Shearer

**Affiliations:** ^1^Department of Biochemistry and Molecular Biology, Cumming School of Medicine, University of CalgaryCalgary, AB, Canada; ^2^Department of Medical Genetics, Cumming School of Medicine, University of CalgaryCalgary, AB, Canada; ^3^Department of Pediatrics, Cumming School of Medicine, University of CalgaryCalgary, AB, Canada; ^4^Department of Clinical Neurosciences, Cumming School of Medicine, University of CalgaryCalgary, AB, Canada; ^5^Department of Physiology and Pharmacology, Cumming School of Medicine, University of CalgaryCalgary, AB, Canada; ^6^Faculty of Kinesiology, University of CalgaryCalgary, AB, Canada

**Keywords:** ketogenic diet, nutrient sensing, mitochondrial dynamics, mitochondrial respiration, liver metabolism, mitochondrial fission, mitochondrial fusion

## Abstract

The ketogenic diet (KD) has been utilized as a dietary therapeutic for nearly a century. One experimental model particularly responsive to the KD is the BTBR^T+tf/j^ (BTBR) mouse, which displays phenotypic characteristics of autism spectrum disorder (ASD) and insulin resistance. Recently, the study of impaired mitochondrial function has become a focal point of research investigating the pathophysiology of ASD. As highly dynamic organelles, mitochondria undergo constant fluctuations in morphology, biogenesis, and quality control in order to maintain cellular homeostasis. An important modifier of mitochondrial dynamics is energy availability. Therefore, the aim of this study was to examine the impact of a KD on mitochondrial dynamics in the liver and brain (prefrontal cortex) of the BTBR mouse model of ASD. Juvenile male C57Bl/6 (B6) and BTBR mice were age-matched to 5 weeks of age before being fed standard chow (CD, 13% kcal fat) or a KD (75% kcal fat) for 10–14 days. Analysis of brain tissue identified differences in mitochondrial gene expression but no correlation with protein levels. Unlike in the brain, KD led to decreased levels of mitochondrial proteins in the liver, despite increased gene expression. Consistent with decreased mitochondrial proteins, we also observed decreased mtDNA for all mice on the KD, demonstrating that the KD reduces the total amount of mitochondria in the liver. In order to explain the discrepancy between protein levels and gene expression, we investigated whether mitochondrial turnover via mitophagy was increased. To this end, we examined expression levels of the mitophagy regulator BNIP3 (BCL2/adenovirus E1B 19 kd-interacting protein 3). BNIP3 gene and protein expression were significantly elevated in liver of KD animals (*p* < 0.05), indicating the potential activation of mitophagy. Therefore, consumption of a KD exerts highly tissue-specific effects, ultimately increasing mitochondrial turnover in the liver, while gene and protein expression in the brain remaining tightly regulated.

## Introduction

The ketogenic diet (KD) has been utilized as a dietary therapy in clinical settings since the early 1920's and is well-documented in mitigating symptoms for a number of diseases, including epilepsy (Rho, 2015), autism spectrum disorder (ASD; Ruskin et al., 2013), and diabetes (Feinman et al., 2014). Mechanistically, the KD functions to minimize carbohydrate availability and favor fatty acid oxidation. Ketone bodies, the by-product of this metabolic shift, are synthesized in the liver and ultimately consumed by major organ systems such as the brain, heart, and skeletal muscle as a major fuel source (Kossoff et al., 2009). Although the mechanisms of ketone body generation and utilization are well-understood, the tissue-specific impact of KD metabolism has yet to be examined.

Responsible for a host of metabolic processes including cellular bioenergetics and energy balance regulation, mitochondria represent the metabolic endpoint for dietary foodstuffs. As the principal site of fatty acid metabolism through β-oxidation, assessment of mitochondrial function through oxidative phosphorylation (OXPHOS) efficiency is a primary measure in KD interventions (Gano et al., 2014; Vidali et al., 2015). However, the literature primarily focuses on the relationship between the KD and brain tissue (Veech, 2004; Masino and Rho, 2012; Milder and Patel, 2012), failing to examine the tissue responsible for ketogenesis—the liver.

In addition to their role in metabolism, mitochondria are known to exist as dynamic organelles. The study of mitochondrial dynamics refers to a group of specialized processes governing mitochondrial morphology, biogenesis, movement, and quality control (Ni et al., 2015; Wai et al., 2016). Mitochondrial morphology can be divided into two aspects: fission (fragmentation) and fusion (elongation). Many proteins and pathways are implicated in mitochondrial fission and fusion although the main regulators of fission are the proteins; dynamin-related protein 1 (DRP1), mitochondrial fission 1 protein (FIS1), mitochondrial fission factor (MFF) and mitochondrial dynamics proteins of 49 and 51 kDa (MiD49/51), whereas fusion is primarily regulated by the proteins; mitofusin 1 & 2 (MFN1, MFN2) and optic atrophy 1 (OPA1; Okamoto and Shaw, 2005; Chan, 2006; Detmer and Chan, 2007; Losón et al., 2013; Hoppins, 2014). Recently, peroxisome proliferator-activated receptor-γ coactivator 1-α (PGC-1α) has emerged as a potential upstream regulator of fission and fusion machinery. PGC-1α governs mitochondrial biogenesis (Dabrowska et al., 2015) and acts by regulating mitochondrial protein translation in response to energy balance fluctuations. Furthermore, variations in nutrient availability can also trigger mitochondrial movement within the cell (Ahmad et al., 2014).

Another important aspect of mitochondrial dynamics is quality control. Termed mitophagy, this mechanism is responsible for the selective lysosome-dependent autophagy of mitochondria (Zhang, 2013). Through the breakdown of dysfunctional mitochondria, mitophagy helps to maintain mitochondrial homeostasis within the cell (Youle and Narendra, 2011). Mitophagy inducing receptors are present on the outer mitochondrial membrane and facilitate lysosomal degradation of mitochondria by localizing microtubule-associated protein 1A/1B-light chain 3 to the outer mitochondrial membrane (Tanida et al., 2008). A common mitophagy regulator is BCL2/adenovirus E1B 19 kd-interacting protein 3 (BNIP3; Ney, 2015). Once triggered, BNIP3 migrates to the outer mitochondrial membrane and is responsible for inducing mitophagy under conditions of cell stress, including hypoxia (Zhang et al., 2008) and fasting (Glick et al., 2012). Interestingly, the KD acts by metabolically mimicking the fasted state and mitophagy regulation has yet to be examined following this therapy.

To this end, we examined the impact of the KD on mitochondrial dynamics in the liver and brain (prefrontal cortex) using two mouse genotypes, the C57Bl/6 (B6) and the BTBR^T+tf/j^ (BTBR). The BTBR mouse was chosen as it has been widely utilized as a model of ASD (Meyza et al., 2013; Newell et al., 2016) and insulin resistance (Stoehr et al., 2000), as well as exhibiting behavioral alterations (Ruskin et al., 2013). Moreover, the KD has recently been shown to alter the metabolome and microbiome of BTBR animals, which are strongly correlated with host metabolic function (Klein et al., 2016; Newell et al., 2016). We hypothesized that the metabolic shift initiated by the KD would involve alterations in the expression of proteins governing mitochondrial dynamics in both the liver and brain.

## Materials and methods

### Animals and dietary interventions

All experimental protocols for this study were completed under the ethics report (A13-0313) approved by the University of Calgary Animal Care and Use Committee, as well as guidelines established by the Canadian Council on Animal Care. Juvenile male C57Bl/6 (B6) and BTBR mice (*n* = 21 and 25, respectively) were separated by genotype and housed 4–6 per cage. Animals were age-matched to 5 weeks of age before being randomly selected for implementation of a standard chow (CD; 13% kcal fat) or ketogenic diet (KD; 75% kcal fat; Bio-Serv F3666, Frenchtown, USA). This resulted in four treatment groups: B6-Chow, B6-Ketogenic, BTBR-Chow, and BTBR-Ketogenic (*n* = 11, 10, 15, and 10, respectively). Prior to sacrifice, animals were housed in a humidity-controlled room with a 12-h light/dark Zeitgeber cycle and were fed *ad libitum*. After 10–14 days of dietary intervention mice were weighed and whole blood was analyzed for glucose and circulating ketone bodies (β-hydroxybutyrate) with Precision Xtra meters (Abbott Laboratories, Alameda, USA) before being sacrificed by cervical dislocation. Liver, brain (prefrontal cortex), and whole blood were removed and either kept fresh or flash-frozen and stored at −80°C until later testing. At the time of sacrifice, animals were 6 weeks of age.

### Mitochondrial respirometry

Fresh liver tissue from the upper left lobe was rinsed and homogenized in mitochondrial isolation buffer [70 mM sucrose, 210 mM mannitol, 5 mM HEPES, 1 mM EGTA, and 0.5% (w/v) fatty-acid free bovine serum albumin (BSA), pH 7.2 at 4°C; Sigma-Aldrich, Oakville, Canada] before undergoing differential centrifugation, according to procedures modified from a previously published report (Rogers et al., 2011). Protein quantification of liver mitochondria was performed with BSA as the standard using the Bradford method (Bio-Rad, Hercules, USA). High-resolution, high-throughput respirometry measures were performed on isolated mitochondrial samples using the XF^e^24 Extracellular Flux Analyzer (Seahorse Bioscience, Billerica, USA) as previously described (Rogers et al., 2011). A total of 8 μg of liver mitochondria was used and each sample was run in triplicate with results normalized to starting protein concentration. Substrate additions were as follows (final concentration): 4 mM ADP, 2 μg/mL oligomycin, 2 μM FCCP, and 2 μM antimycin A (Sigma-Aldrich). Respiration buffer contained 5 mM succinate and 2 μM rotenone (Sigma Aldrich) in order to isolate mitochondrial respiration through Complex II of the electron transport chain.

### Citrate synthase enzyme activity

Citrate synthase activity, a mitochondrial enzyme and marker of mitochondrial content, was measured from liver homogenates using a spectrophotometric method (Tweedie et al., 2011). Enzyme activity was then normalized to starting protein concentration.

### RNA extraction and qRT-PCR

Total ribonucleic acid (RNA) was extracted from 25 mg of frozen liver and brain tissues using the PureLink RNA Mini Kit (Life Technologies, Burlington, Canada) and was measured using a NanoDrop-1000 (Thermo Fisher Scientific, Burlington, Canada). Reverse transcription was performed with 1 μg of RNA using the iScript cDNA Synthesis Kit (Bio-Rad). cDNA products were quantified using a NanoDrop-1000 (Thermo Fisher Scientific). qRT-PCR was performed in 20 μl reaction volumes using 50 ng of complimentary DNA (cDNA). Primer pairs were synthesized by the University of Calgary Core DNA Services (Table S1). The PCR conditions were as follows: 95°C—2 min, 40 cycles of (95°C—30 s, 60°C—30 s, 72°C—30 s), 72°C—2 min. Samples were run in triplicate on the same reaction plate using the CFX96 Real-Time PCR Detection System (Bio-Rad) with β-actin as a loading control. Data analysis was performed in accordance with previously published work using the 2^−ΔΔCt^ method (Lee et al., 2012).

### Ratio of mtDNA/nDNA copy number

As surrogates for mitochondrial abundance through measurements of relative mtDNA copy numbers, liver and brain tissues were used to determine the mtDNA/nDNA ratio for the mitochondrial genome-encoded cytochrome *b* (MT-CYB) and NADH dehydrogenase 2 (ND2). The expression of these genes was then expressed in relation to the nuclear-encoded β-globin. The relative mtDNA copy number was measured according to the method of Wong and Cortopassi (2002) and Liu et al. (2003).

### Protein immunoblotting

Liver and brain tissues were homogenized in a lysis buffer containing 20 mM NaCl, 20 mM Tris-HCl, 0.1 mM EDTA, 1% Triton X-100, 0.5% sodium deoxychloate, and 0.1% β-mercaptoethanol along with a protease inhibitor cocktail (Sigma-Aldrich) and a phosphatase inhibitor cocktail (Thermo Fisher Scientific). Tissue homogenates then underwent protein quantification with BSA as the standard using the Bradford method (Bio-Rad). Liver and brain lysates in Laemmli buffer were separated by SDS-PAGE. Samples were loaded onto 4–12% Bis-Tris SDS-polyacrylamide gels (Invitrogen) and then transferred using gel electrophoresis to polyvinylidine difluoride membranes (Millipore, Billerica, USA). Membranes were probed with primary antibodies overnight at 4°C on a shaking platform and then incubated with secondary antibodies for 1.5 h at room temperature. Primary antibodies were as follows: DRP1 (catalog no. ab56788, Abcam, Cambridge, USA), FIS1 (catalog no. sc-98900, Santa Cruz Biotechnologies, Dallas, USA), MFF (catalog no. ab81127, Abcam), MFN1 (catalog no. ab57602, Abcam), MFN2 (catalog no. ab101055, Abcam), OPA1 (catalog no. ab42364, Abcam), BNIP3 (catalog no. 3769, Cell Signaling Technology, Whitby, Canada), PGC-1α (catalog no. sc-13067, Santa Cruz), mitochondrial outer membrane protein—voltage-dependent anion-selective channel 1 (VDAC1; catalog no. ab15895, Abcam), Total OXPHOS (catalog no. ab110413, Abcam), and β-actin (catalog no. ab8226, Abcam). Membranes were exposed to the chemiluminescent SuperSignal West Femto Maximum Sensitivity Substrate (Life Technologies) and imaged using the ChemiGenius imaging system (Syngene, Frederick, USA). Densitometry was performed using GeneTools (Syngene) with β-Actin (Abcam) used as a loading control.

### Statistical analyses

Statistical analysis was performed using IBM SPSS Statistics for Windows, Version 21.0. Differences between the genotype and diet were determined by a two-way analysis of variance (ANOVA), followed by a Tukey's *post-hoc* test where *p* < 0.05 was considered to be significant. Data are expressed as mean ± *SEM*.

## Results

### Animal characteristics

Animal weight, blood glucose, and blood ketone levels are reported in Table 1. Consuming the KD caused diet-dependent reductions in body mass across both genotypes (*p* < 0.05). BTBR mice also exhibited a greater body weight than B6 (*p* < 0.05). Mice fed a KD resulted in significantly elevated circulating ketone bodies and decreased blood glucose levels across both genotypes (*p* < 0.05). Animal characteristics have been previously reported (Klein et al., 2016), and they are included here to provide a frame of reference for the mitochondrial function data in the BTBR mouse.

**Table 1:** **Animal characteristics**.

	**B6**		**BTBR**	
	**Chow**	**Ketogenic**	**Chow**	**Ketogenic**
Mass (g)	18.4±0.8^*^	10.5±0.3	28.6±1.3^*^^†^	15.9±1.0^†^
Blood glucose (mmol/L)	10.4±0.6^*^	4.3±0.5	7.8±0.3^*^	3.7±0.5
Blood ketones (mmol/L)	0.9±0.1	5.1±0.8^*^	0.9±0.1	5.1±0.8^*^

*Age-matched B6 and BTBR mice were sacrificed following 10–14 days of either standard chow or ketogenic feeding. All characteristics were assessed prior to sacrifice. Data represent mean ± SEM (B6-Chow, B6-Ketogenic, BTBR-Chow, and BTBR-Ketogenic; n = 11, 10, 15, and 10, respectively)*.

* *Indicates a difference due to diet at p < 0.05*.

† *Indicates a difference due to genotype at p < 0.05*.

### Mitochondrial function

**Figure 1:**
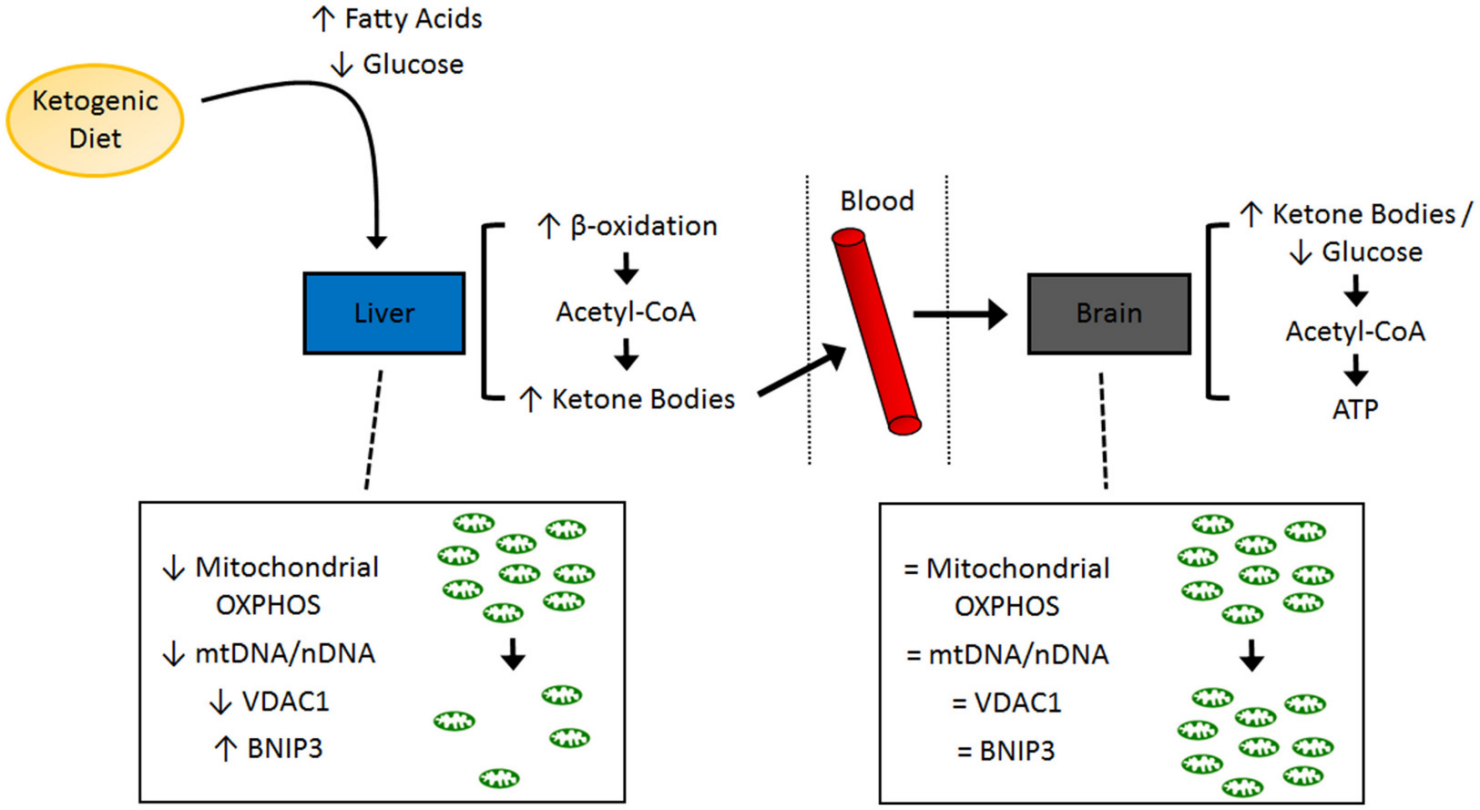
**Schematic of molecular pathways investigated in the present study**. A summary of the tissue-specific findings related to mitochondrial function and mitochondrial dynamics. ATP, adenosine triphosphate; BNIP3, BCL2/adenovirus E1B 19 kd-interacting protein 3; OXPHOS, oxidative phosphorylation; VDAC1, voltage-dependent anion-selective channel 1.

A general schematic of experimental findings is shown in Figure 1.

Liver mitochondrial respiration is described in Table 2. ADP facilitated respiration (basal respiration) was lower in B6-Ketogenic mice compared to all other groups (*p* < 0.05). FCCP (maximal respiration) and oligomycin (proton leak) mediated respiration decreased in B6 animals fed a KD (*p* < 0.05). Antimycin A respiration (non-mitochondrial respiration) showed no significant differences between either dietary treatment or genotype (*p* > 0.05). There were no differences in citrate synthase enzyme activity across groups.

**Table 2:** **Mitochondrial respirometry performed on mitochondria isolated from liver tissue homogenates using an XF^**e**^24 Extracellular Flux Analyzer**.

	**B6**		**BTBR**	
	**Chow**	**Ketogenic**	**Chow**	**Ketogenic**
ADP	202±8.6^*^	139±12	165±24	150±16
Oligomycin	79±23^*^	37±8.3	64±10	56±9.3
FCCP	215±13^*^^†^	55±14	174±29	152±19
Antimycin A	19±10	17±5.1	41±15	18±9.2
Citrate synthase activity	34±1.0	35±2.6	34±1.4	36±2.1

*Age-matched B6 and BTBR mice were sacrificed following 10–14 days of either chow or ketogenic diet feeding. Respiration buffer contained 5 mM succinate and 2 μM rotenone in order to isolate mitochondrial respiration through Complex II of the electron transport chain. Oxygen consumption rate was normalized to protein concentration, resulting in each value expressed as pmoles/min/μg mitochondrial protein. The mitochondrial enzyme citrate synthase is listed as a marker of mitochondrial abundance; each value is expressed as μM/min/mg protein. Data represent the mean ± SEM (B6-Chow, B6-Ketogenic, BTBR-Chow, and BTBR-Ketogenic; n = 11, 10, 15, and 10, respectively)*.

* *Indicates a difference due to diet at p < 0.05*.

† *Indicates a difference due to genotype at p < 0.05*.

### Protein immunoblotting of mitochondrial OXPHOS subunits

**Figure 2:**
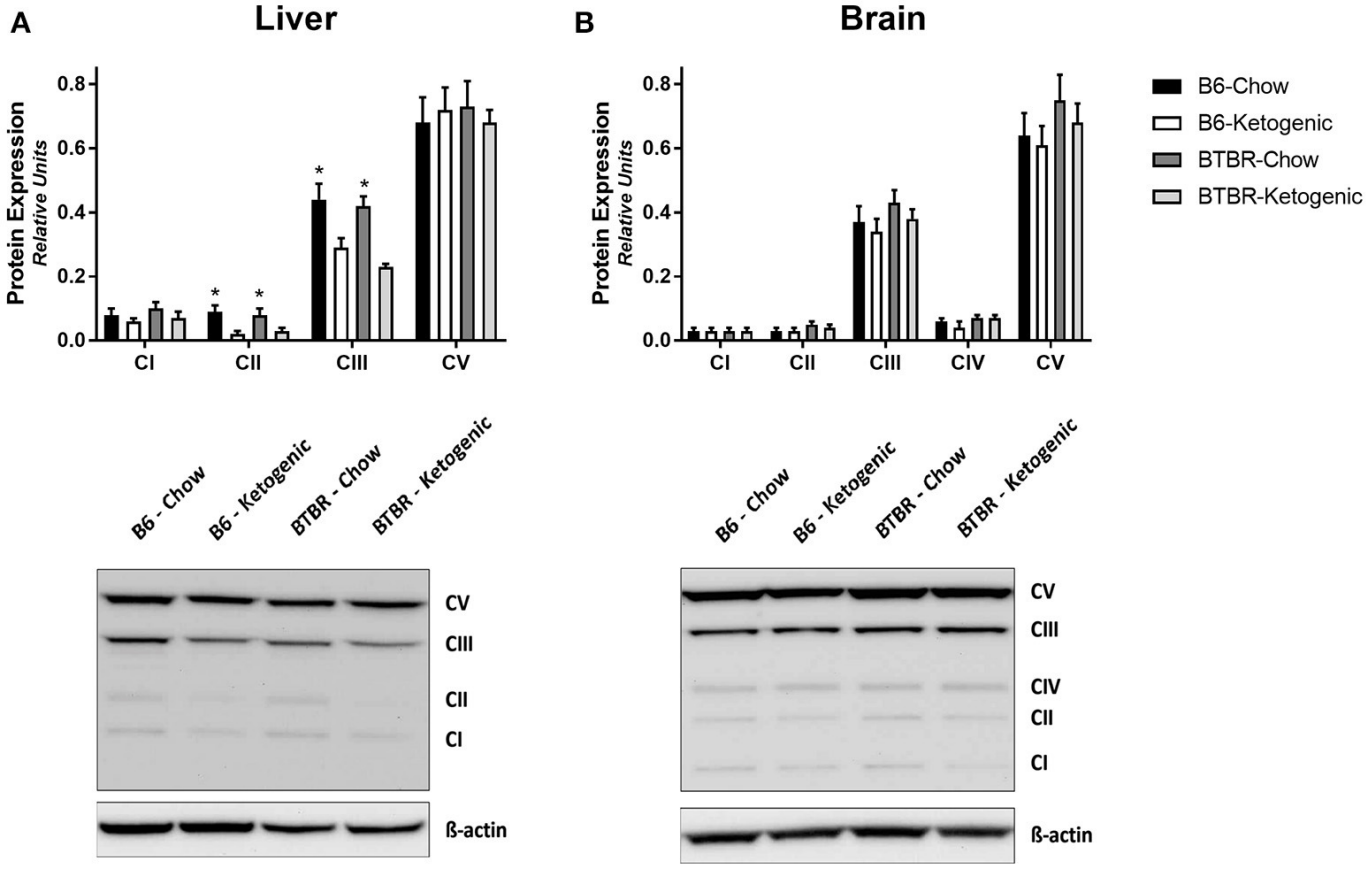
**Quantification of protein immunoblots for mitochondrial electron transport system subunits**. Liver **(A)** and brain **(B)** tissues were analyzed for protein expression of mitochondrial oxidative phosphorylation (OXPHOS) subunits (CI–V). All protein expression data was analyzed using β-Actin as a loading control. All data are presented as mean ± *SEM* (B6-Chow, B6-Ketogenic, BTBR-Chow, and BTBR-Ketogenic; *n* = 8 for each). ^*^Indicates a difference due to diet at *p* < 0.05.

Protein levels of mitochondrial electron transport system subunits are reported in Figure 2. Brain tissues demonstrated no variation due to genotype or dietary intervention (Figure 2B). However, KD fed mice had decreased Complex II and Complex III protein expression in liver (Figure 2A).

### Mitochondrial dynamics. gene expression of mitochondrial morphology regulators (qRT-PCR)

**Figure 3:**
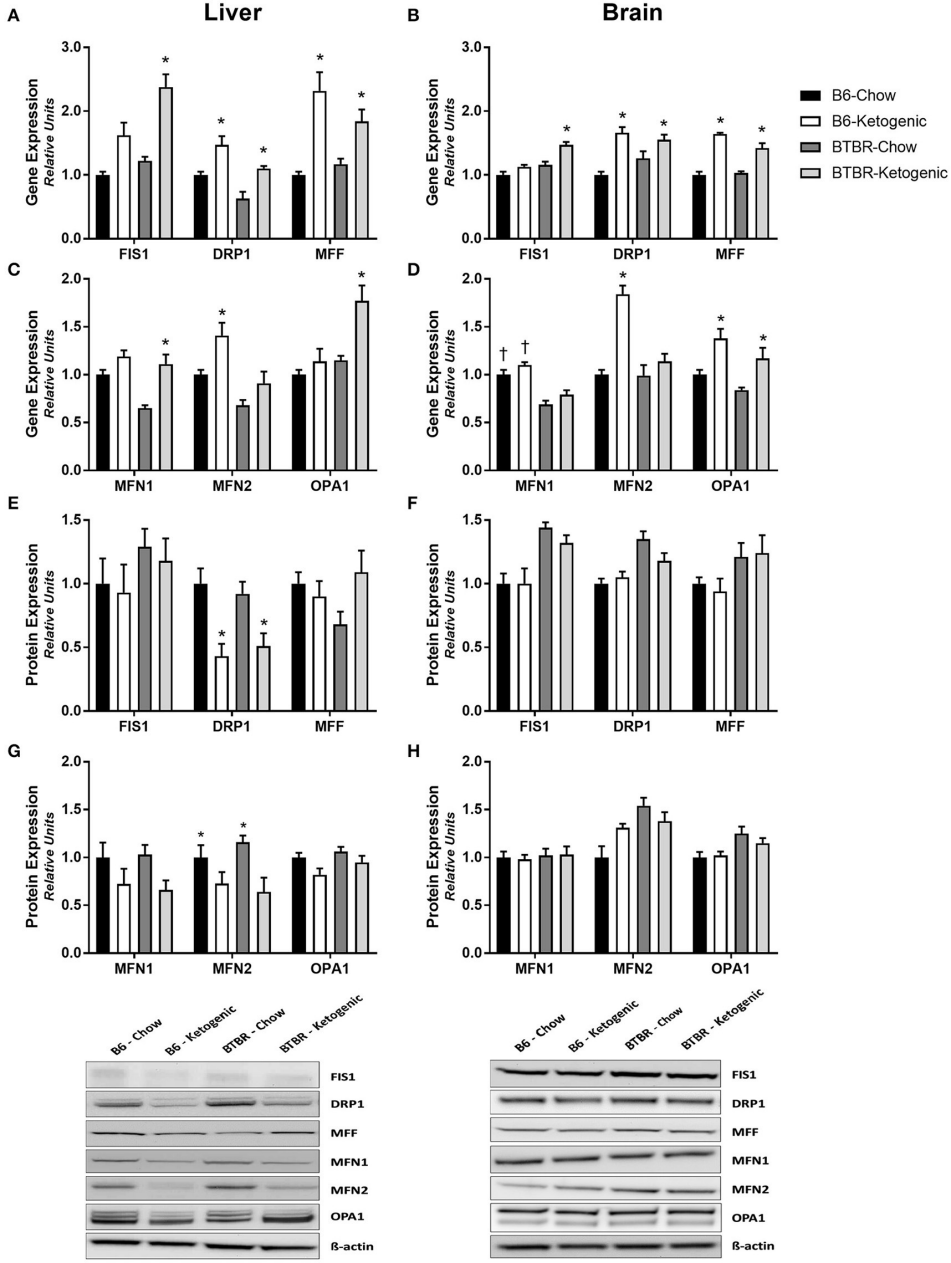
**Assessment of mitochondrial morphology regulators employing gene and protein expression**. Liver and brain tissues were analyzed for gene (qRT-PCR) and protein expression (protein immunoblotting) of key mitochondrial morphology mediators. **(A)** Mitochondrial fission gene expression measured in liver. **(B)** Mitochondrial fission gene expression measured in brain. **(C)** Mitochondrial fusion gene expression measured in liver. **(D)** Mitochondrial fusion gene expression measured in brain. **(E)** Expression of mitochondrial fission proteins measured in liver. **(F)** Expression of mitochondrial fission proteins measured in brain. **(G)** Expression of mitochondrial fusion proteins measured in liver. **(H)** Expression of mitochondrial fusion proteins measured in brain. All gene expression data were collected using qRT-PCR with β-Actin as a loading control. All protein expression data were analyzed using β-Actin as a loading control. All data are presented as mean ± *SEM* (B6-Chow, B6-Ketogenic, BTBR-Chow, and BTBR-Ketogenic; *n* = 8 for each). ^*^Indicates a difference due to diet at *p* < 0.05. ^†^Indicates a difference due to genotype at *p* < 0.05.

The gene expression of key mitochondrial fission and fusion mediators was examined in both the liver and brain (Figure 3). KD consumption resulted in wide-spread increases in gene expression across both genotypes. Both liver and brain tissues had elevated expression of key fission (FIS1, DRP1, and MFF) and/or fusion (MFN1, MFN2, and OPA1) genes following KD (Figures 3A–D; *p* < 0.05).

### Protein immunoblotting of mitochondrial morphology regulators

Interestingly, no differences in protein expression for any of the same key mitochondrial fission or fusion mediators was found in brain (Figures 3F,H). In contrast, the protein expression for these mitochondrial fission and fusion mediators in liver were found to decrease following KD (Figures 3E,G). Specifically, expression of the fission protein DRP1 and the fusion protein MFN2 were decreased after KD treatment, while the remaining proteins trended toward a decline after KD consumption.

### Mitochondrial abundance and biogenesis

**Figure 4:**
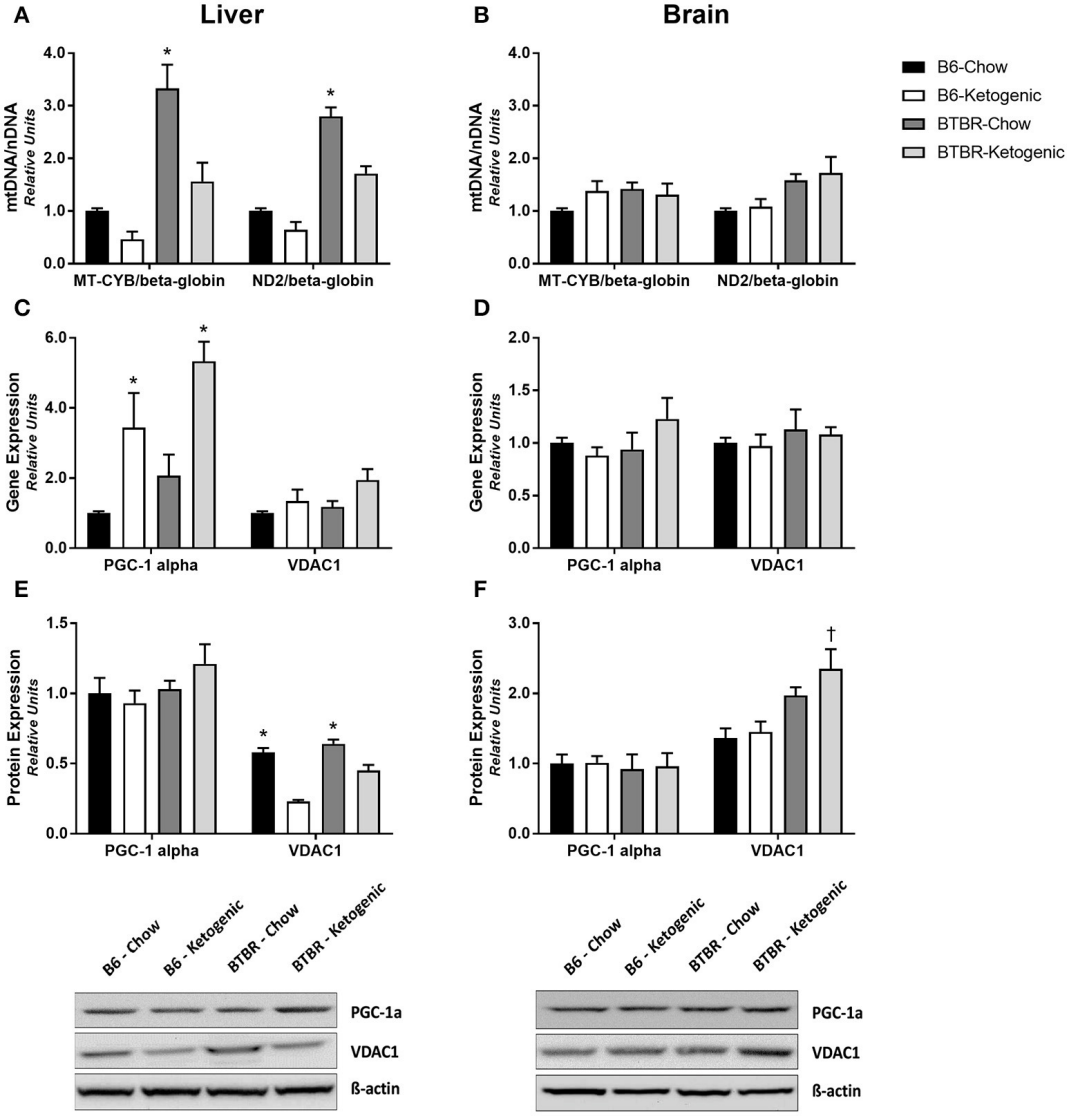
**Evaluation of mitochondrial gene and protein expression to infer regulation of mitochondrial abundance and biogenesis**. Liver and brain tissues were analyzed to measure the mtDNA/nDNA ratios for the mitochondrial genome-encoded cytochrome *b* (MT-CYB) and NADH dehydrogenase 2 (ND2), expressed relative to the nuclear-encoded β-globin. **(A)** mtDNA/nDNA ratio measured from liver. **(B)** mtDNA/nDNA ratio measured from brain. Liver and brain tissues were also analyzed for gene and protein expression of the mitochondrial biogenesis regulator—peroxisome proliferator-activated receptor-γ coactivator 1-α (PGC-1α) and the marker of mitochondrial abundance—voltage-dependent anion-selective channel 1 (VDAC1). **(C)** Gene expression of PGC-1α and VDAC1 measured in liver. **(D)** Gene expression of PGC-1α and VDAC1 measured in brain. **(E)** Protein expression of PGC-1α and VDAC1 measured in liver. **(F)** Protein expression of PGC-1α and VDAC1 measured in brain. All qRT-PCR data were collected with β-Actin as a loading control and are presented as mean ± *SEM* (B6-Chow, B6-Ketogenic, BTBR-Chow, and BTBR-Ketogenic; *n* = 8 for each). ^*^Indicates a difference compared to all other groups at *p* < 0.05. ^†^Indicates a difference due to genotype at *p* < 0.05.

Expectedly, no changes in the mtDNA/nDNA copy number ratio were detected in brain tissue (Figure 4B). By contrast, dietary differences were identified within each genotype for liver. BTBR mice consuming a KD resulted in a significant decrease in the mtDNA/nDNA copy number ratio in the liver (Figure 4A; *p* < 0.05). A similar diet-induced trend was identified in B6 mice although this was not statistically significant. Additionally, measurements of gene and protein expression for mitochondrial biogenesis regulator—PGC-1α and mitochondrial outer membrane protein—VDAC1, we also performed. Once again, analysis of brain tissue proved unremarkable (Figures 4D,F) with only protein expression of VDAC1 being elevated in BTBR-Ketogenic mice when compared to either B6 fed group. Liver tissue, however, yielded an increase in PGC-1α gene expression following KD consumption (Figure 4C; *p* < 0.05). Interestingly, protein expression for PGC-1α was not responsive in a similar manner, and this correlated with a significant decrease in VDAC1 protein expression for KD mice (Figure 4E; *p* < 0.05).

### Mitochondrial quality control (mitophagy)

**Figure 5:**
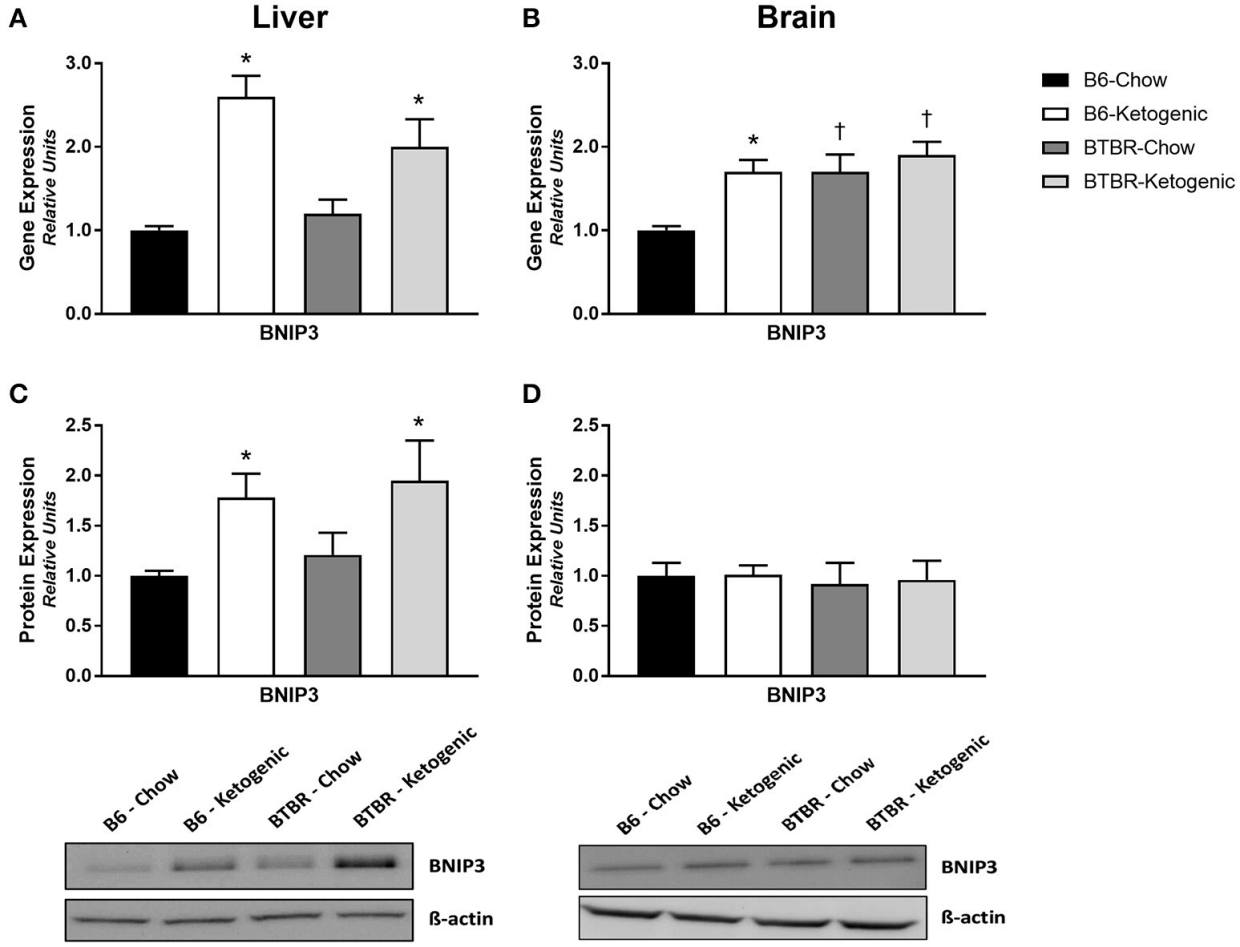
**Determination of mitochondrial gene and protein expression for mitochondrial quality control regulator BNIP3**. Liver and brain tissues were analyzed for gene (qRT-PCR) and protein expression (protein immunoblotting) of the mitophagy promoter—BCL2/adenovirus E1B 19 kd-interacting protein 3 (BNIP3). **(A)** Gene expression of BNIP3 measured in liver. **(B)** Gene expression of BNIP3 measured in brain. **(C)** Protein expression of BNIP3 measured in liver. **(D)** Protein expression of BNIP3 measured in brain. All data were analyzed using β-Actin as a loading control and are presented as mean ± *SEM* (B6-Chow, B6-Ketogenic, BTBR-Chow, and BTBR-Ketogenic; *n* = 8 for each). ^*^Indicates a difference due to diet at *p* < 0.05. ^†^Indicates a difference due to genotype at *p* < 0.05.

Examination of the mitophagy promoter, BNIP3, revealed unremarkable gene and protein expression profiles in brain (Figures 5B,D). Although gene expression of BNIP3 was elevated in all groups when compared to B6-Chow (*p* < 0.05), no differences in protein expression were identified. Comparatively, analysis of liver tissue proved interesting as both gene and protein expression were upregulated in B6 and BTBR mice following KD treatment (Figures 5A,C; *p* < 0.05).

## Discussion

Previous work has shown that the KD modulates metabolic, cognitive, and behavioral impairments in a number of disease states (Evangeliou et al., 2003; Herbert and Buckley, 2013). Although the mechanism(s) of action through which the KD imparts its beneficial effects are yet to be elucidated, we sought to examine the impact of the KD on mitochondrial dynamics in B6 and BTBR mice, a model known to be highly responsive to the KD (Ruskin et al., 2013). To this end, we examined mitochondrial function and aspects of mitochondrial dynamics in the liver and brain of B6 and BTBR mice fed either a CD or KD. The major findings of this study are as follows: (1) KD feeding results in unremarkable changes to mitochondrial function or mitochondrial dynamics in the brain; (2) KD consumption spurs alterations in mitochondrial function in liver tissue; (3) the KD significantly changes gene and protein expression for regulators of mitochondrial morphology, abundance, and biogenesis in the liver; (4) the KD results in elevated gene and protein expression of the mitophagy regulator BNIP3.

Responsible for β-oxidation of fatty acids, liver mitochondrial function is crucial to healthy energy balance, and a pivotal site of metabolism when consuming a KD. However, the vast majority of existing literature on the KD has focused on the brain due to its well-documented neurologic benefits (Veech, 2004; Masino and Rho, 2012; Milder and Patel, 2012). Both liver and brain tissues were therefore assayed for mitochondrial respiratory rates, although technical issues enabled data acquisition for only liver. In order to focus on fatty acid metabolism involvement, respiratory substrates succinate, and rotenone were used. This allowed us to analyze mitochondrial bioenergetics of Complex II (Schönfeld et al., 2010). Although a limitation of the present work is the constraint in tissue availability, future research also warrants respiration data for Complex I substrates. Potentially leading to differences in mitochondrial function, examination of mitochondrial OXPHOS protein levels was performed. Although brain tissue showed no differences, the liver of KD fed mice from both genotypes demonstrated lower Complex II and Complex III mitochondrial protein expression (Figure 2A). This reduction in KD animals is likely due to hepatic fat accumulation, inefficient mitochondrial bioenergetics, and insulin resistance. However, the effects on mitochondrial respiration were only observed in B6 and not BTBR animals. This is likely a result of “two hits,” or simultaneous metabolic insults in the BTBR model. Originally studied as a model of insulin resistance (Flowers et al., 2007), the BTBR mouse demonstrates a tempered metabolic disturbance to the KD diet rich in fatty acids compared to B6 mice. In its early stages, insulin resistance results in augmented mitochondrial bioenergetics and enzyme activity—but the continued insult having the opposite effect and eventually led to impaired mitochondrial function and dysregulated mitochondrial dynamics in the liver (Putti et al., 2015). Therefore, there were likely early mitochondrial adaptations in the BTBR mouse stemming from a pre-disposition to insulin resistance and as a result, the impact of the KD on mitochondrial respiration was less pronounced.

Through mimicking the fasted state, the KD induces ketogenesis in the liver, with the resultant ketone bodies being metabolized by the brain. As such, a unique relationship between liver and brain metabolism exists during KD treatment. We hypothesized that this shift in nutrient availability would impact mitochondrial dynamics. Thus, we examined differences in gene and protein expression in the regulation of mitochondrial morphology. Existing research supports the notion that fission gene expression is upregulated with nutrient availability (Holmström et al., 2012; Jheng et al., 2012) and fusion genes are upregulated with nutrient deficiency (Rambold et al., 2011). Contrary to this, the present liver and brain data suggest a global upregulation of fission and fusion genes when fed the KD. These findings may be linked to tissue-specific metabolic fluctuations resulting from the stress of excess liver β-oxidation and brain ketone body metabolism with the KD. However, examination of protein expression suggests no changes in brain and a global downregulation in the liver following KD feeding. This indicates a tissue specific sensitivity to changes in sources of energy. The differences between gene and protein in brain are likely due to the tight regulation of energy production, in order to maintain normal brain function (Du et al., 2008). However, gene and protein differences in liver allow for rapid metabolic adaptations should carbohydrates become available. Unfortunately, literature examining the role of the KD to impact mitochondrial dynamics is limited. Interestingly, existing work using co-cultured cells with ketone body supplementation in the growth medium (Santra et al., 2004) and mtDNA deletor mice consuming a KD for over 30 weeks (Ahola-Erkkila et al., 2010), have demonstrated positive effects of the KD on host mitochondrial function. However, stark differences in duration of diet and type of model organism make it difficult to compare those results to the present findings.

One explanation for the disparity between gene expression and protein levels is increased turnover of mitochondria via mitophagy. Although microscopic evidence of differences in mitochondrial mass and morphology are the current gold-standard, tissues from this animal cohort were not prepared at the time of sacrifice. Therefore, we first surveyed for differences in mitochondrial abundance by comparing the ratio of mtDNA/nDNA, which assesses the relative copy number abundance and therefore infers the amount of mitochondria present in a sample. No differences were detected in the brain; conversely, the mtDNA/nDNA copy number was decreased in the liver of KD mice, irrespective of genotype. In order to confirm these findings, we then investigated gene and protein expression for VDAC1—a commonly studied protein to assess mitochondrial abundance (Fritz et al., 2013). Once again there were no notable differences in the brain. In contrast, VDAC1 protein expression was decreased by the KD in liver. These findings explain, in part, the decreased Complex II and Complex III OXPHOS protein levels in KD-fed liver tissue. Decreases in mitochondrial abundance caused by dysfunctional energy homeostasis have been linked to increased mitochondrial turnover (Youle and Narendra, 2011). This may indicate why the mitochondrial abundance in liver, whose energy balance is affected, declines while the mitochondrial abundance in brain remains unchanged.

In order to identify whether the KD impacts mitophagy signaling in liver or brain, we assessed levels of BNIP3—a primary mitophagy regulator. Not surprisingly, there were no differences in BNIP3 levels noted in the in brain; however, BNIP3 protein and gene expression were both elevated in liver of both genotypes after KD consumption. These results suggest that upregulation of BNIP3-induced mitophagy in the liver may succeed the KD in order to sequester mitochondrial disturbances spurred by impairments in energy balance. Initially identified as exclusively pro-apoptotic, BNIP3-induced mitophagy actually lowers the cell's capacity to release cytochrome c and preserves cell viability (Zhu et al., 2013). This provides a clear connection between the decreases in mtDNA/nDNA copy number and both VDAC1 and OXPHOS protein expression in liver. However, as mitophagy involves a diverse number of proteins and other organelles, the complexities of this process must be further studied in response to the KD.

The KD has been shown to cause elevated PPARα—the transcription factor responsible for regulating fatty acid metabolism in the liver (Kimura et al., 2013), and PGC-1α—a primary coactivator responsible for regulation of mitochondrial biogenesis (Liang and Ward, 2006). During conditions of energy deprivation, PPARα promotes the metabolic conversion of fatty acids to ketone bodies through upregulation of β-oxidation (Braissant et al., 1996). Activation of PPARα facilitates the downstream upregulation of BNIP3, which is required to prevent excess lipid accumulation in liver tissue (Glick et al., 2012), culminating in increased mitophagy, and decreased mitochondrial abundance. As the upstream coactivator of PPARα, PGC-1α responds to physiologic and pathologic stimuli by regulating energy metabolism. Previous work examining the metabolic impact of the KD has shown that liver gene expression of PGC-1α is elevated in mice consuming a KD, although protein expression was not examined (Jornayvaz et al., 2010). Liver tissues of KD mice in the present study also yield elevated gene expression of PGC-1α, although no changes in protein levels were found. Our data demonstrate that both B6 and BTBR mice respond to the KD by increasing gene and protein expression of mitophagy regulator BNIP3 in the liver. These findings are consistent with decreased mtDNA levels, decreased VDAC1 levels and the increases in PGC-1α gene expression. Increasing rates of mitophagy in the liver may also explain the discrepancy between gene and protein levels of the mitochondrial dynamics regulators investigated in the present study. As the metabolic pathways involving PGC-1α and PPARα are vastly complex, the specific roles of individual gene and protein alterations must be delineated accordingly. This suggests that although PGC-1α is a primary upstream regulator of both PPARα and BNIP3, the mechanism of how the KD influences the regulation of BNIP3-induced mitophagy involves various gene and protein interactions, which in turn will require further studies into mitochondrial morphology and dynamics.

In summary, this study provides novel information about the relationship between the KD and two key tissues, the liver and brain. Our results show that the brain experiences no disturbances in mitochondrial dynamics, likely due to tight regulation of gene and protein expression. However, the liver withstands the burden of metabolizing the excess lipids consumed during the KD and experiences widespread changes in mitochondrial function and mitochondrial dynamics. This acts as a pathway to provide energy and to preserve brain function, which is supplied through ketone body production by the KD. Given the involvement of dysfunctional mitochondria in various disease states and the use of the KD as a clinical intervention, understanding how energy intake affects mitochondrial dynamics may lead to insights into how the KD exerts its broad-spectrum clinical benefits.

## Author contributions

JS, TS, YA, and JR designed and developed the research. CN, TS, YA, DH, AK, JR, and JS conducted experiments, collected, and analyzed data. CN and JS wrote the manuscript and all authors read and approved the final manuscript.

### Conflict of interest statement

The authors declare that the research was conducted in the absence of any commercial or financial relationships that could be construed as a potential conflict of interest.
