# Supplementary material for: Tissue Specific Impacts of a Ketogenic Diet on Mitochondrial Dynamics in the BTBRT+tf/j Mouse
Source: Front Physiol. 2016 Dec 27;7:654. doi: 10.3389/fphys.2016.00654 (PMC5186794; doi:10.3389/fphys.2016.00654)
Supplement: Supplementary file 1 [file Table1.DOCX]

**S1 Table.** Mitochondrial and nuclear primers used for qRT-PCR

| **Gene** | **Forward Primer (5'-3')** | **Reverse Primer (5'-3')** |
| --- | --- | --- |
| DRP1 | GCGCTGATCCCGCGTCAT | CCGCACCCACTGTGTTGA |
| FIS1  MFF | GCCCCTGCTACTGGACCAT  TGGCAGTGAGTTCTGAGGTG | CCCTGAAAGCCTCACACTAAGG  AATCCCTACGAACACTTGGG |
| MFN1 | TCTCCAAGCCCAACATCTTCA | ACTCCGGCTCCGAAGCA |
| MFN2 | ACAGCCTCAGCCGACAGCAT | TGCCGAAGGAGCAGACCTT |
| OPA1 | TGACAAACTTAAGGAGGCTGTG | CATTGTGCTGAATAACCCTCAA |
| ND2 | CTCCTGCCTCTGTGGTTGAG | CCGATTGGATGAAGTCGACA |
| MT-CYB | GCCACCTTGACCCGATTCT | TTCCTAGGGCCGCGATAAT |
| BNIP3  VDAC1  PGC-1α | GCTCCCAGACACCACAAGAT  GCTAAGGATGACTCGGCTTTAAGG  GCTTTCTGGGTGGACTCAAGT | TGAGAGTAGCTGTGCGCTTC  AGGTTAAGTGATGGGCTAGGATGG  TCTAGTGTCTCTGTGAGGACTG |
| β-actin | ACGGCCAGGTCATCACTATTC | AGGAAGGCTGGAAAAGAGCC |
